# Members of the Oral Microbiota Are Associated with IL-8 Release by Gingival Epithelial Cells in Healthy Individuals

**DOI:** 10.3389/fmicb.2017.00416

**Published:** 2017-03-15

**Authors:** Katharina Schueller, Alessandra Riva, Stefanie Pfeiffer, David Berry, Veronika Somoza

**Affiliations:** ^1^Department of Nutritional and Physiological Chemistry, Faculty of Chemistry, University of ViennaVienna, Austria; ^2^Research Network “Chemistry Meets Microbiology”, University of ViennaVienna, Austria; ^3^Department of Health Sciences, Università degli Studi di MilanoMilan, Italy; ^4^Division of Microbial Ecology, Department of Microbiology and Ecosystem Science, University of ViennaVienna, Austria; ^5^Christian Doppler Laboratory for Bioactive Aroma Compounds, University of ViennaVienna, Austria

**Keywords:** healthy humans, inflammation, IL-8, nutrition, oral microbiota

## Abstract

The triggers for the onset of oral diseases are still poorly understood. The aim of this study was to characterize the oral bacterial community in healthy humans and its association with nutrition, oral hygiene habits, and the release of the inflammatory marker IL-8 from gingival epithelial cells (GECs) with and without stimulation by bacterial endotoxins to identify possible indicator operational taxonomic units (OTUs) associated with inflammatory marker status. GECs from 21 healthy participants (13 females, 8 males) were incubated with or without addition of bacterial lipopolysaccharides (LPSs), and the oral microbiota was profiled using 16S rRNA gene-targeted sequencing. The basal IL-8 release after 6 h was between 9.9 and 98.2 pg/ml, and bacterial communities were characteristic for healthy oral microbiota. The composition of the oral microbiota was associated with basal IL-8 levels, the intake of meat, tea, white wine, sweets and the use of chewing gum, as well as flossing habits, allergies, gender and body mass index. Additionally, eight OTUs were associated with high basal levels of IL-8 and GEC response to LPS, with high basal levels of IL-8, and 1 with low basal levels of IL8. The identification of indicator bacteria in healthy subjects with high levels of IL-8 release is of importance as they may be promising early warning indicators for the possible onset of oral diseases.

## Introduction

Over the last decade, understanding of the development and progression of oral diseases as well as their prevention and treatment has continuously improved (Petersen, 2003; Touger-Decker and Mobley, 2013). In addition to personal oral hygiene and the availability of dental care, nutrition has been increasingly acknowledged to be an important factor affecting health status of the oral cavity (Touger-Decker and Mobley, 2013). Certain nutritional patterns have been associated with an increased risk of developing oral diseases, such as consumption of sugar-rich food and drinks, whereas the consumption of protein-containing foods such as meat, cheese or fish, as well as use of chewing gum have been associated with a decreased risk (Touger-Decker and Mobley, 2013). The oral microbial community has recently come into focus as an important aspect of oral health care as the type of bacteria present in the oral cavity has a major impact on dental health status (Darveau, 2010; Dentino et al., 2013). 16S rRNA gene sequencing of the human oral bacterial community has shown that dental plaque consists of over 500 different species, dominated by the phyla *Firmicutes*, *Bacteroidetes*, *Proteobacteria*, *Actinobacteria*, *Spirochaetes*, and *Fusobacteria* (Wade, 2013). The commensal microbiota plays an important role in maintaining oral and systemic health.

The commensal oral microbiota inhibits colonization by opportunistic pathogens, a phenomenon known as colonization resistance (Wade, 2013). Because all surfaces of the mouth are colonized by commensals, there are few binding sites available for pathogens. Some health-associated bacteria have been shown to be antagonistic to oral pathogens. For example, *Streptococcus salivarius* strain K12 produces a bacteriocin which inhibits the growth of gram-negative species that are associated with periodontitis and halitosis *in vitro* (Wade, 2013). Socransky et al. (1998) showed that the presence of specific bacterial species in human plaque samples form reoccurring complexes that may be important in blocking pathogen colonization. An important step in understanding the microbiology of periodontitis was the observation that during the development of disease, a shift in those microbial complexes occurs (Darveau, 2010). While the microbiota present at healthy sites are mainly comprised of gram-positive species (*Streptococcus* spp.), at diseased sites different bacteria occur, such as gram-negative, anaerobic species including PG*, Tannerella forsythia, Prevotella* ssp., and *Treponema denticola* (Darveau, 2010; da Silva-Boghossian et al., 2011).

Lipopolysaccharides from the outer membranes of bacteria can activate TLRs, thus triggering an innate immune response (Hans and Hans, 2011). The LPS of the oral pathogens PG and EC differ in their chemical structure and immune-modulating properties (Tamura et al., 1992). Recent studies in our group have shown that the immune response to PG- and EC-LPS in *in vitro* challenged human gingival fibroblasts, as well as in immune cells such as monocytes and macrophages, is characterized by a 20–200-fold increase of inflammatory chemokines and cytokines within 6 h (Ehrnhöfer-Ressler et al., 2013; Walker et al., 2013; Soares et al., 2014; Schueller et al., 2015). While elevated levels of cytokines and chemokines have also been detected in diseased sites of patients suffering from gingivitis and severe periodontitis, the innate immune response to plaque bacteria in healthy tissues is normally delicately balanced (i.e., host homeostasis) to maintain healthy tissues and low levels of inflammatory markers (Bartold and Narayanan, 2006; Darveau, 2010). In periodontitis, bacterial communities are shifted and pathways of host homeostasis are altered, leading to an inflammatory infiltrate containing migrating macrophages and activated fibroblasts and endothelial cells as well as increased levels of inflammatory markers (Bartold and Narayanan, 2006). The chemokine IL-8 has important functions in oral health by facilitating the transit of activated immune cells into and through gingival tissues, and promoting immune-cell adhesion, tissue remodeling, and angiogenesis (Campbell et al., 2013). IL-8 is elevated in the saliva of oral cancer patients (720 vs. 250 pg/ml for control individuals) and has been proposed as a biomarker for the development of oral cavity and oropharyngeal squamous cell carcinoma (St. John et al., 2004). In patients with severe periodontitis, 4000–5500 pg/μl IL-8 have been detected in crevicular fluid at non-diseased sites (Goutoudi et al., 2012).

While considerable research has been done on the differences in inflammatory markers and bacterial communities for defining stages of oral disease progression, so far, no phylotypes or indicator indicator OTUs have been defined in oral health that precede the first clinical symptoms of oral disease. As most studies on periodontal health and disease have been done in a comparative way, in this study we deliberately only included orally healthy individuals to examine pre-clinical differences. Also, clinical studies mostly use tissues from tooth extractions or crevicular fluid for determining the inflammatory status, whereas in this study, we determined the release of basal IL-8 over 6 h from GECs in *ex vivo* incubations. Additionally, the impact of 6 h *ex vivo* stimulation of GECs with two different kinds of LPS (PG and EC) on IL-8 levels was assessed. The aim of this study was to link basal release levels of IL-8, nutrition, personal oral hygiene and the bacterial community in individuals with healthy gums, and to identify indicator OTUs for an immune response to external bacterial stimuli, established by measurement of IL-8 release from GECs. Our findings present new insights into the differences in IL-8 levels and the oral microbiota in healthy individuals and the response of GECs to external bacterial stimuli, which will be useful for future studies of oral health. This is, to our knowledge, the first study identifying indicator OTUs among oral bacteria associated with high basal release levels of IL-8 from GECs.

## Materials and Methods

All chemicals and reagents were purchased from Sigma-Aldrich (Vienna, Austria), unless otherwise stated.

### Study Population and Questionnaire

Twenty one healthy participants (13 females, 8 males) between the ages of 22 and 55 were recruited for the study. Exclusion criteria were pregnancy, smoking, and antibiotic therapy in the last 3 months. Participants signed an informed consent and were asked to complete a questionnaire including general information, eating and drinking habits, as well as personal oral hygiene and medication prior to enrolling in the study (**Table 1**). All participants self-reported to be free of oral diseases.

**Table 1:** Factors considered in the questionnaire.

General	Nutrition	Oral hygiene	Medication
Age	Tea^a,b^	Toothpaste	Hormones^g,^*^∗^*
Height	Coffee^a^	Mouthwash^f,^*^∗^*	Pain killers^h,^*^∗^*
Weight	Meat^c^	Flossing^f,^*^∗^*	
Gender	Beer^c^	Chewing gum^f,^*^∗^*	
Allergies^∗^	Red wine^d^		
	White wine^d^		
	Fruits^e^		
	Sweets^c^		

^a^*Per day [L];*

^b^*black, green, and herbal tea;*

^c^*per week [g] or [L];*

^d^*per month [L];*

^e^*portions per week;*

^f^*>1 per week;*

^g^*oral contraceptives, hormone replacement therapy;*

^h^*oral intake in the last 30 days;*

^∗^*categories yes/no.*

### Collection of GECs and Bacteria

Participants were asked to abstain from brushing their teeth and eating breakfast in the morning of GEC collection. Material (cells and bacteria) were collected by the participants themselves by thoroughly brushing the upper and lower outer gums using three sterile brushes (PraxisDienst, Longuich, Germany). Brushes were then rinsed in 7.2 ml of DMEM containing 10% fetal bovine serum (FBS, Thermo Scientific, Waltham, MA, USA) and 8 mM Glutamine. After thoroughly re-suspending the collected material, 1 ml of suspension was centrifuged at 16,000 × *g* for 7 min at 4°C and the supernatant was removed. The pellet was then flash-frozen in liquid nitrogen and kept at -80°C until DNA extraction.

### *Ex vivo* Incubation of GECs

Epithelial cells were counted, the cell suspension aliquoted into three 15 ml tubes, centrifuged for 5 min at 400 × *g* at room temperature and re-suspended in fresh medium to a final concentration of 150,000 living cells/ml, as determined by trypan blue exclusion test. Two different LPS (EC-stock 1 mg/ml ddH_2_O from Sigma-Aldrich and PG-stock 1 mg/ml ddH_2_O, euBio, Vienna, Austria) were added to the respective aliquots for final concentrations of 10 μg/ml LPS. The third aliquot was left without any addition for determination of basal levels of IL-8. GECs were seeded to a concentration of 150,000 living cells/ml in 24-well plates and were incubated for 6 h in triplicate in an incubator at 37°C and 5% CO_2_. After incubation, pH measurements of all three incubations were done with pH strips, and the mean pH of three measurements was used for analysis (interval 0.5 units).

### Measurement of IL-8

After 6 h incubation, supernatants of the 24-well plates were collected, centrifuged at 16,000 × *g* for 10 min at 4°C and two aliquots per well were stored at -80°C further analysis. Basal IL-8 levels after 6 h cultivation under standard conditions (control), IL-8 levels after incubation with 10 μg/ml EC-LPS for 6 h (ecLPS) and IL-8 levels after incubation with 10 μg/ml PG-LPS for 6 h (pgLPS) were determined in the supernatant by ELISA (Merck Millipore, Darmstadt, Germany), following the manufacturer’s instructions.

### DNA Extraction, Preparation of 16S rRNA Gene Target Amplicons Libraries, and Sequence Analysis

DNA was extracted from samples using a phenol–chloroform protocol with bead-beating and precipitated with 0.1 volume of 3 M Na-Acetate and 0.6 volumes of ice-cold isopropanol (Griffiths et al., 2000). DNA was subjected to a two-step PCR amplification targeting the 16S rRNA gene using the forward primer S-D-bact-0341-b-S-17 (5′-CCTACGGGNGGCWGCAG-3′) and the reverse primer S-D-bact-0785-a-A-21 (5′-GACTACHVGGGTATCTAATCC-3′). The first PCR reaction was performed in triplicate with 25 cycles. The PCR products were then pooled and submitted to a second step PCR of 10 cycles with the addition of 8 nt sample-specific barcode sequence (Herbold et al., 2015). The barcoded amplicons were purified after the second step with ZR-96 DNA Clean-up Kit (Zymo Research, USA) and quantified using the Quant-iT PicoGreen dsDNA Assay (Invitrogen, USA). An equimolar library was constructed by pooling samples, and the resulting library was sequenced on the Illumina MiSeq platform at Microsynth AG (Balgach, Switzerland). Sequence data were sorted into libraries using the 8 nt sample-specific barcode and primer using a custom-made in-house script, quality-filtered according to the Earth Microbiome Project guidelines, and paired-end reads were concatenated (Bokulich et al., 2012). Reads were then clustered into species-level OTUs of 97% sequence identity, checked for chimeras using USEARCH, and taxonomically classified using the Ribosomal Database Project näive Bayesian classifier (Wang et al., 2007). Sequence data has been deposited in the NCBI Sequence Read Archive under SRP075956.

### Statistical Data Analysis

#### IL-8 Data Analysis

Data were analyzed using SigmaPlot 11.0 (SystatSoftware, USA). Correlations of basal IL-8 values and metric questionnaire variables were done with Spearman Rank correlations, comparisons of two groups were done using Student’s *t*-test. In case of not normally distributed data, comparisons between two groups were done using Mann–Whitney Rank Sum test. A *p*-value ≤ 0.05 was considered significant. For analysis of the response to stimulation with LPS, participants with relative IL-8 values lower than 100% (as determined by LPS-treated over basal IL-8 levels) after stimulation were designated as “non-responders,” while participants with relative IL-8 values over 100% after stimulation with LPS were designated as “responders.”

#### Microbiota Analysis

Statistical analysis was performed using the statistical software R^1^. To avoid biases related to uneven library depth, sequencing libraries were subsampled to a number of reads smaller than the smallest library (4,500 reads). Significant factors were evaluated using permutational multivariate analysis of variance (perMANOVA) and ordination was performed using RDA in the vegan package in R version 1.17-4 (Oksanen et al., 2010). Alpha diversity metrics were calculated using the vegan package and statistical differences in alpha diversity was performed using the Student’s *t*-test. Variables were expressed as mean ± SD and a *p*-value ≤ 0.05 was considered significant. Indicator OTU analysis was carried out using the indicspecies package (De Caceres and Legendre, 2009).

## Results

### Basal IL-8 Levels

**FIGURE 1:**
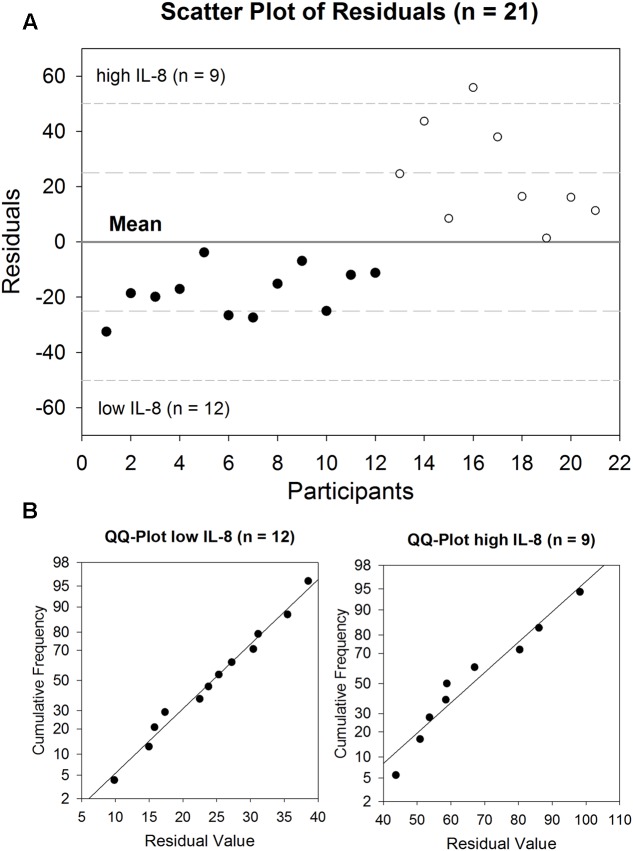
**Basal release of IL-8 by GECs after 6 h incubation (range: 9.9–98.2 pg/ml, mean: 42,4 pg/ml) depicted as a (A)** scatter plot of residuals of all participants (*n* = 21). The solid line represents the mean value, the medium dashed line the specification level of mean ± 1 SD, the short dashed line the specification level of mean ± 2 SD. **(B)** QQ-Plots for low IL-8 group (*n* = 12) and high IL-8 group (*n* = 9).

*Ex vivo* incubated GECs showed a release of IL-8 into the supernatant between 9.9 and 98.2 pg/ml after 6 h, with a mean value of 42.4 pg/ml for all participants (*n* = 21, tr = 3). In order to gain further insight into the differences in basal IL-8 release, while maintaining sufficient group sizes for indicator OTUs analysis, participants were divided into two groups with *n* = 12 (designated ‘low IL-8’) and *n* = 9 (designated ‘high IL-8’), based on the residual analysis, as determined by positive and negative residual values (**Figure 1A**). Both groups were normally distributed as revealed by Shapiro–Wilk normality testing (P_lowIL-8_ = 0.974, P_highIL-8_ = 0.525) and QQ-plots (**Figure 1B**).

### Correlations of Basal IL-8 and Metric Questionnaire Data

Statistical analysis for the questionnaire data was done with all participants (*n* = 21). The correlation of basal IL-8 levels with all metric parameters of the questionnaire (**Table 2**) showed a positive correlation with the pH of the supernatant (*R* = 0.477, *p* = 0.029), as well as the consumption of red wine (*R* = 0.457, *p* = 0.037) and white wine (*R* = 0.485, *p* = 0.026).

**Table 2:** Spearman correlations of basal IL-8 levels with metric questionnaire parameters considering all participants (*n* = 21).

IL-8 vs.	Corr. Coeff.	*p*-value
Age	0.104	0.649
BMI	-0.0286	0.899
pH	0.477	0.029^∗^
Tea	0.275	0.223
Coffee	-0.109	0.633
Meat	-0.0939	0.682
Beer	0.0305	0.895
Red wine	0.457	0.037^∗^
White wine	0.485	0.026^∗^
Fruits	0.107	0.637
Sweets	-0.150	0.509

*A p-value ≤ 0.05 was considered significant, and is shown with an asterisk*.

### Pairwise Comparison of Basal IL-8 and Nominal Questionnaire Data

For pairwise comparison, toothpaste ingredients were differentiated (no/yes) by the addition of zinc, film formers (methyl vinyl ether/maleic anhydride copolymer) and plant extracts (*chamomilla*, *mentha*, *salvia*, *myrrha*, *krameria triandra*). Pairwise comparison of basal IL-8 levels with nominal data for all participants (*n* = 21) showed no differences in basal IL-8 levels concerning gender, allergies, the use of floss, mouthwash, the consumption of chewing gum, the intake of hormones (only women) and pain killers (**Table 3**).

**Table 3:** Analysis of basal IL-8 levels regarding nominal questionnaire data.

Factor (no/yes)	Group sizes (*p*-value)
Gender (f/m)	13 vs. 8 (0.447)
Allergies	13 vs. 8 (0.980)
Toothpaste ingredients	
Zinc	12 vs. 9 (0.382)
Plant extracts	17 vs. 4 (0.046^∗^)
Film formers	15 vs. 6 (0.791)
Mouthwash	13 vs. 8 (0.262)
Flossing	8 vs. 13 (0.753)
Chewing gum	9 vs. 12 (0.386)
Tea	13 vs. 8 (0.139)
White wine	10 vs. 11 (0.032^∗^)
Red wine	12 vs. 9 (0.082)
Beer	9 vs. 12 (0.370)
Hormones	5 vs. 8 (0.195)
Pain killers	8 vs. 13 (0.525)

*Additionally, the factors tea, white wine, red wine and beer were also categorized with yes/no, and sample sizes of compared groups (Student’s *t*-test, Mann–Whitney Rank Sum test) are stated in parentheses next to calculated *p*-values; a *p*-value ≤ 0.05 was considered significant, and is shown with an asterisk*.

The presence of zinc and film formers in the toothpaste reported by participants did not have an influence on basal IL-8 levels, but for plant extracts, pairwise comparison resulted in *p* = 0.046, with mean values of 47.6 ± 24.5 pg/ml (*n* = 17) in the absence, and 20.2 ± 12.6 pg/ml (*n* = 4) in the presence of plant extracts in the toothpaste, respectively. Using a nominal dataset for the consumption of tea, beer, white and red wine (no or yes), a difference was found between white wine drinkers, and non-white wine drinkers (*p* = 0.032), with mean values of basal IL-8 for non-white wine drinkers (*n* = 10) and white wine drinkers (*n* = 11) of 29.5 ± 13.7 and 54.1 ± 27.8 pg/ml, respectively. The observed significant correlation in basal IL-8 levels for red wine consumption (**Table 2**) did not occur after nominal categorization of data (*p* = 0.082).

### The Oral Microbiota Composition

**FIGURE 2:**
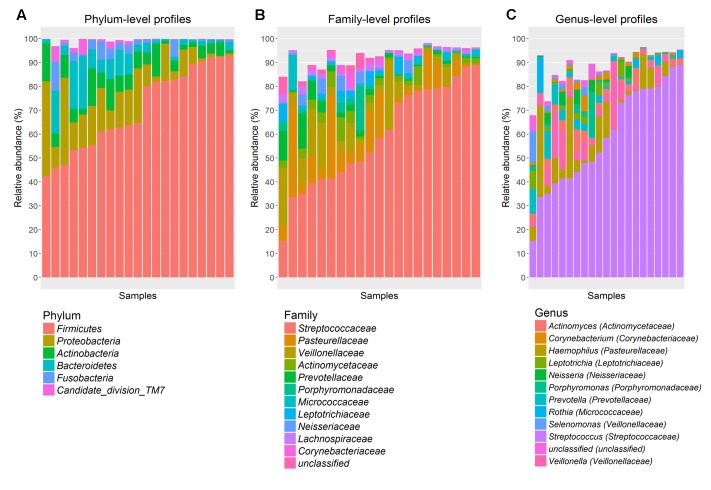
**Abundant bacterial taxa from gum brushes. (A)** phylum-, **(B)** family-, and **(C)** genus-level taxon profiles are shown. Abundant taxa, defined as having a mean relative abundance of >1%, are shown.

The predominant bacterial phyla (in % abundance; mean ± SD) in gum brushes were *Firmicutes* (70.5 ± 17.2), followed by *Proteobacteria* (12.2 ± 10.7), *Actinobacteria* (6.6 ± 4.5), *Bacteroidetes* (6.1 ± 6.5), *Fusobacteria* (2.5 ± 3.1) and Candidate division TM7 (1.1 ± 1.9) (**Figure 2A**). The most dominant family detected was *Streptococcaceae* (59.3 ± 21.3) (**Figure 2B**), which was represented at the genus level by *Streptococcus* (59.3 ± 21.3) (**Figure 2C**).

### Alpha Diversity

Oral microbiota richness was not significantly different between low and high basal IL-8 levels (observed species: *p* = 0.534; Chao1 estimated richness: *p* = 0.163). Likewise, alpha diversity metrics, which consider both community richness and evenness, were not significantly different (Shannon: *p* = 0.449; inverse Simpson *p* = 0.895).

### Associations of Oral Microbiota with Nutrition and Oral Hygiene Habits

**FIGURE 3:**
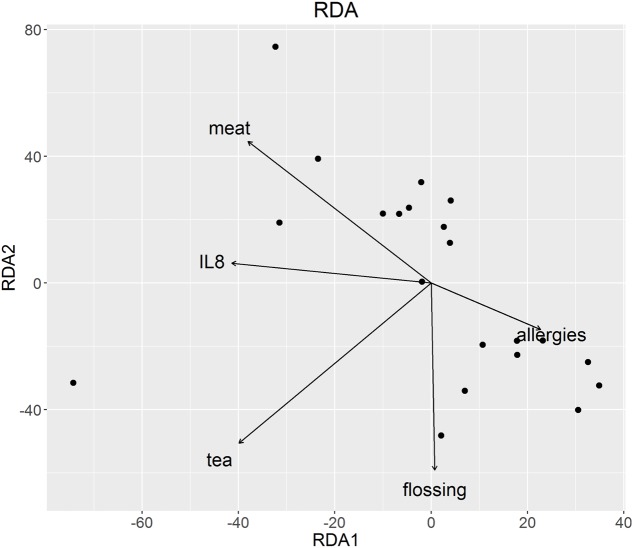
**Redundancy analysis of oral microbiota at OTU level**. Parameters significant at OTU level in the perMANOVA analysis were plotted and are represented by arrows. The direction of the arrows shows the association between variables, in this case with meat and IL-8.

The composition of the oral microbiota was associated with nutrition and oral hygiene habits at the OTU level as well as taxonomic levels from genus to phylum, with significant associations with basal IL-8 levels, gender, body mass index (BMI), allergies, flossing habits, chewing gum use, and consumption of tea, meat, beer, white wine, and sweets (perMANOVA; *p* < 0.05, **Table 4**). We performed RDA considering these significant parameters to visualize these associations. RDA ordination revealed a strong correlation between basal IL-8 levels and meat consumption, but not for tea, floss habits and allergies (**Figure 3**).

**Table 4:** Non-parametric multivariate analysis of variance (perMANOVA) analysis performed at every taxonomical level.

Parameters	Taxonomical level	*p*-value
IL-8	OTU	0.016
Tea	OTU Phylum Family	0.007 0.001 0.041
Meat	OTU Phylum	0.005 0.018
Allergies	OTU	0.033
Flossing	OTU Phylum	0.010 0.015
Gender	OTU Phylum	0.058 0.014
BMI	Phylum Family	0.005 0.050
Beer	Phylum	0.007
White wine	Phylum Family Genus	0.001 0.009 0.022
Sweets	Phylum	0.041
Chewing gum	Phylum	0.007

*Only significant nutritional and oral hygiene habits are shown*.

### Stimulation of Oral GECs with 10 μg/ml LPS from EC or PG

*Ex vivo c*hallenge of brushed cells with EC- or PG-LPS did not affect basal IL-8 levels, with means for untreated cells, ecLPS and pgLPS-incubated cells of 42.4 ± 25.0, 39.2 ± 22.3, and 40.7 ± 24.1 pg/ml, respectively (**Table 5**). Also, for the earlier designated low IL-8 (*n* = 12) and high IL-8 (*n* = 9) groups, no differences in absolute basal IL-8 levels of controls compared to levels after treatment with EC- and PG-LPS were found. In the low IL-8 group, the absolute mean values for non-treated cells, ecLPS- and pgLPS-exposed cells were 24.4 ± 8.8, 24.1 ± 8.5, and 24.2 ± 9.6 pg/ml, respectively. Absolute means for high IL-8 group were 66.4 ± 18.1, 59.3 ± 18.5, and 62.8 ± 19.2 pg/ml for non-treated cells, ecLPS and pgLPS exposed cells, respectively. On relative levels (ecLPS/pgLPS over basal IL-8 in percent), differences to control (100%) were found in the high level ecLPS group, with an unexpected decrease in IL-8 levels of -10.6 ± 16.1% (*p* = 0.038), whereas no other group showed significant alterations in IL-8 release levels. The here obtained *ex vivo* results of bacterial challenge differ from literature results of *in vitro* experiments, as elaborated in the introduction, and warrant further studies to verify and elucidate the effect. To prevent over-interpretation of data, release data after challenge with EC-LPS were excluded from indicator OTU analysis.

**Table 5:** *p*-values for pairwise comparisons of relative [LPS/basal IL-8 %] levels for all participants (*n* = 21), low IL-8 (*n* = 12), and high IL-8 (*n* = 9) groups after *ex vivo* stimulation of oral GECs with EC LPS (10 μg/ml) or PG LPS (10 μg/ml) for 6 h.

	IL-8 relative [LPS/basal IL-8 %]	
	ecLPS *p*-value	pgLPS *p*-value
All participants (*n* = 21)	0.405	0.788
Low (<40 pg/ml, *n* = 12)	0.478	0.478
High (>40 pg/ml, *n* = 9)	0.038^∗^	0.178

*A p-value ≤ 0.05 was considered significant, and is shown with an asterisk. No pairwise comparisons of absolute IL-8 levels were statistically significant*.

To analyze whether the response to the *ex vivo* stimulation of GECs with EC-LPS and PG-LPS was dependent on basal IL-8 levels, data within the low and high IL-8 groups were divided into two categories (non-responders and responders) and a Fisher’s exact test was done with the resulting counts for non-responders and responders (contingency table). This analysis resulted in a *p*-value of 0.065.

### Indicator OTUs Based on IL-8 Levels and PG LPS-Induced IL-8 Response in GECs

**FIGURE 4:**
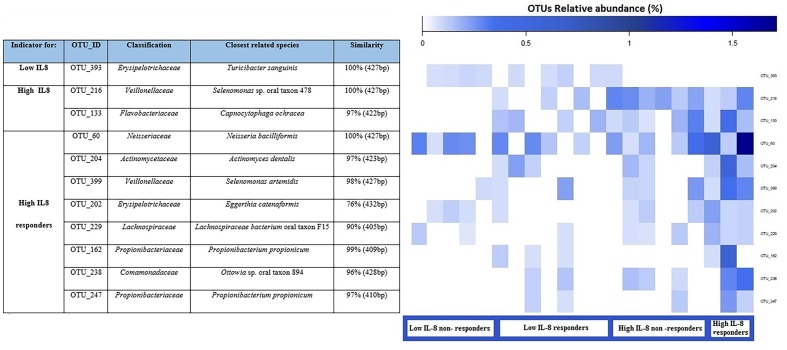
**Dominant indicator OTUs are presented for pgLPS response with their relative abundance (as percent) for each sample**. Each OTU is annotated with the closest reference sequence and the sequence similarity with the reference species. The indicator OTUs are grouped according to the condition for which they are an indicator for each combination. Each column of the heatmap represents a single sample.

We used an indicator OTU analysis to determine the strength of the association between OTU abundances and the levels of basal IL-8 release (low IL-8, high IL-8) combined with responder/non-responder status from LPS challenge (expressed as LPS-induced IL-8/basal IL-8 in percent). We considered the abundances of individual OTUs and determined indicator OTUs for the following groups: low IL-8, high IL-8 (irrespective of response to LPS challenge), as well as low IL-8 non-responders to PG-LPS, low IL-8 responders to PG-LPS, high IL-8 non-responders to PG-LPS, and high IL-8 responders to PG-LPS. Statistically significant indicators for either single groups or combinations of groups were then identified. We identified 11 indicator OTUs in total: one for “low IL-8,” two for “high IL-8,” and eight for “high IL-8 responders.” **Figure 4** shows an elevated abundance of indicator OTUs in the high IL-8 group, in particular for high IL-8 responders. OTU_216 (*Veillonellaceae*) and OTU_60 (*Neisseriaceae*) were the most abundant indicators for high IL-8 status.

## Discussion

This study aimed at establishing associations of nutrition, oral hygiene habits and basal release of IL-8 from GECs with the oral bacterial community to identify indicator OTUs that might predict onset of oral pathologies. Basal IL-8 levels obtained in this study were neither comparable to levels found in crevicular fluid of patients with periodontitis (over 90,000-fold higher), nor to levels obtained by stimulated salivary measurement (250 pg/ml) in healthy individuals, which is to be expected due to impaired oral health and different sampling method as compared to our study (Navazesh and Christensen, 1982; Goutoudi et al., 2012). Comparable to this study, release levels of about 30–50 pg/ml IL-8 were found in primary sterile cultures of fibroblasts and periodontal ligament cells from tooth resections in healthy patients (Scheres et al., 2010). The basal levels of IL-8 in this study were found to positively correlate with pH (**Table 2**) but no significant association between pH and the bacterial community composition was found. Nevertheless, the positive correlation of the pH (values between 5.2 and 8.5) could be related to acidic or basic bacterial and cellular products released into the medium. It has to be considered that although the incubations were done without addition of antibiotics, the culture medium contains buffering salts, thus possibly biasing pH measurements.

In our study, the bacterial community of the mouth was dominated by the phyla *Firmicutes*, followed by *Proteobacteria, Actinobacteria*, *Bacteroidetes*, *Fusobacteria* and *Candidate division* TM7, which is consistent with the oral “core” microbiota reported in previous studies (Zaura et al., 2009; Lazarevic et al., 2010). Nutrition and oral hygiene habits can influence the oral microbiota composition and inflammatory state. Considering the latter, the presence of plant extracts in toothpaste showed an effect on basal IL-8 release (**Table 3**). This is in accordance with literature, as many medicinal plants that are used in toothpastes are anti-inflammatory, anti-bacterial, anti-fungal or astringent (Hotwani et al., 2014). The present analysis found a positive correlation of white and red wine consumption with basal IL-8 levels, and a significant difference between non-white wine drinkers and white wine drinkers (**Tables 2**, **3**). Studies on alcohol consumption and the risk of periodontitis have reported variable findings. Susin et al. (2015) reported that a higher alcohol intake related to the incidence of periodontitis in women, whereas no such correlations could be shown in men (*n* = 1115). On the other hand, Kongstad et al. (2008) found a negative association of alcohol consumption and clinical signs of oral disease in men but not in women (*n* = 1521). In the present study, the separate analysis of gender subdivided by alcohol consumption would results in very small number of participants, which do not allow for such an analysis (*n* = 1–3 for some groups). Interestingly, red wine consumption was also positively correlated with IL-8 levels, as red wine is especially rich in polyphenols, has been shown to attenuate inflammatory markers in gastric inflammation as well as colonic epithelial cells (Mayo et al., 2000; Nunes et al., 2012). Considering our results of healthy participants, it might be hypothesized that moderate consumption of alcohol activates immune pathways in indirectly exposed oral cells, leading to higher basal release levels of IL-8. In our study, associations could be found between the composition of the oral microbiota and the use of chewing gum as well as oral hygiene habits like flossing. It has been demonstrated that the use of xylitol gum decreased the level of *Streptococcus mutans* but did not change the salivary microbial composition (Söderling et al., 2015), and that flossing habits reduce gingival inflammation by removing interproximal plaque (Berchier et al., 2008).

Moreover, we analyzed associations between the composition of the oral microbiota and consumption of different foods and drinks for which an impact on the inflammatory status has been discussed, such as meat, sweets, most commonly consumed alcoholic beverages and tea. Tea polyphenolic catechins, in particular epigallocatechin gallate and epicatechin gallate, can inhibit the growth of a wide range of gram-positive and gram-negative bacterial species with moderate potency. These molecules may be useful in the control of common oral infections, such as dental caries and periodontal disease (Taylor et al., 2005). In particular, catechin gallates inhibit growth and adherence to oral epithelial cells of *P. gingivalis* (Sakanaka et al., 1996) and *Prevotella* (Hirasawa et al., 2002).

As many oral bacteria possess glucose transport systems, they are capable of utilizing dietary carbohydrates and some sugar alcohols. Incorporated carbohydrates and sugar alcohols are subjected to glycolysis via metabolic reactions catalyzed by constitutive and inducible enzymes. Polysaccharides can be hydrolysed into oligosaccharides, disaccharides, and monosaccharides by host and bacterial glycosidases. For example, host α-amylase hydrolyses cooked starch into carbohydrates, which can then be incorporated and metabolized by oral bacteria (Takahashi, 2015). Interestingly, there was a strong association between oral microbiota, meat consumption and basal IL-8 levels (**Figure 3**). The association between oral microbiota and meat may be explained by the degradation activity of proteins into peptides and amino acids by bacterial proteases and peptidases in the oral cavity (Takahashi, 2015).

In established *in vitro* oral and immune cell lines, levels of inflammatory markers are up-regulated after stimulation with LPS from various bacterial strains (Ehrnhöfer-Ressler et al., 2013; Walker et al., 2013; Schueller et al., 2015). This is also true for primary sterile cultures of oral fibroblasts (Nagaoka et al., 1996). In our study, using freshly sampled epithelial cells and not using antibiotics, this kind of up-regulation was not established for IL-8 after 6 h of stimulation with pgLPS. Muthukuru et al. (2005) found that in healthy tissues, the expression of TLRs is much lower than in periodontal disease, which would account for the absence of stimulation by their agonist LPS. Additionally, Scheres and Crielaard (2012) demonstrated that primary sterile gingival fibroblast cultures were desensitized to live PG after repeated contact with the pathogen. In this study, cells were cultured directly after sampling from the mouth, with live plaque bacteria still present. It is therefore very likely that GECs exhibited tolerance to EC- and PG-LPS, as observed in the experiments by Scheres and Crielaard (2012). The significant down-regulation of IL-8 release after stimulation of GECs with a non-oral-derived bacterium (EC) only occurred in the high IL-8 group on relative levels. Muthukuru et al. (2005) demonstrated that a repeated challenge with LPS from EC results in a significant down-regulation of TLRs on the cell-surface, thus impeding or reducing activation of IL-8 transcription and subsequent protein release. No association could be found between basal IL-8 levels and response to LPS challenge (*p* = 0.065). However, the indicator OTU analysis yielded very interesting results (**Figure 4**). The indicator OTUs that we identified are typical members of the oral cavity according to the Human Oral Microbiome Database (HOMD) (Chen et al., 2010) and some have also been associated with periodontal disease or other inflammatory pathologies. For example, *Capnocytophaga ochracea* is present in the dental plaque biofilm of patients with periodontitis and it has been reported in several cases of periodontal disease (Hosohama-Saito et al., 2016). *Propionibacterium propionicum* was found in root canal-treated teeth with apical periodontitis (Siqueira and Rocas, 2009), *Eggerthia catenaformis* was recently isolated in a patient with a dental abscess (Kordjian et al., 2015) and *Actinomyces dentalis* was isolated from pus of a human dental abscess (Hall et al., 2005). In particular, the genus *Actinomyces* houses several long-established human pathogens. Some species cause the inflammatory disease actinomycosis, whereas others are associated with various non-specific inflammatory processes, or may play a role in the development of dental plaque and subsequent caries or periodontal diseases (Hall et al., 2005). Likewise, *Selenomonas* species dominated the diseased sites of subjects with generalized aggressive periodontitis. *Selenomonas sputigena* was the most frequently detected bacterial species whereas other species of *Selenomonas* were often present in high levels, including *Selenomonas noxia, Selenomonas* oral clone EW076, *Selenomonas* oral clone EW084 and *Selenomonas* oral clone CS002. Gonçalves et al. (2012) revealed that *Selenomonas* phylotypes were frequently part of the subgingival microbiota of generalized aggressive periodontitis patients. Also Drescher et al. (2010) analyzed the topography of the subgingival biofilm by fluorescence *in situ* hybridization (FISH) and electron microscopy in subjects with generalized aggressive periodontitis, chronic periodontitis and periodontitis-resistant subject and revealed that *Selenomonas* spp. appeared in large numbers in all parts of the collected biofilms and seemed to make a relevant contribution to their structural organization.

In our study, we found an association between basal IL8 levels and microbiota, suggesting a link between oral bacteria and inflammatory state. Moreover, a link between nutrition, personal oral hygiene with oral microbiota and IL8 levels was also observed. The elevated abundance of some bacterial OTUs in high IL-8 responders, indicating a higher inflammatory activity GEC, may be causally linked to stimulation by bacterial LPS. The indicator OTUs that we identified in healthy subjects may hold promise as biomarkers for inflammatory status of the oral cavity as well as for risk of onset of oral pathologies. The validation of these biomarkers will require further studies with larger cohorts and designs where cohorts are followed until some develop disease so that their prognostic value can be evaluated.

## Ethics Statement

All subjects gave written informed consent in accordance with the Declaration of Helsinki. As samples were anonymized and provided by healthy individuals, ethics committee approval was not required according the regulations of the University of Vienna.

## Author Contributions

KS designed the study, performed experiments and data analysis, and drafted the manuscript. AR performed microbiota experiments and data analysis and drafted the manuscript. SP performed experiments and data analysis. DB performed microbiota data analysis and contributed to manuscript writing. VS designed the study and contributed to manuscript writing.

## Conflict of Interest Statement

The authors declare that the research was conducted in the absence of any commercial or financial relationships that could be construed as a potential conflict of interest.

## Footnotes

1 https://www.r-project.org/

## Abbreviations

EC: *Escherichia coli*
GEC/GECs: gingival epithelial cells
LPS: lipopolysaccharides
OTUs: operational taxonomic units
PG: *Porphyromonas gingivalis*
RDA: redundancy analysis
TLR: toll-like receptors.
